# Efficient Record Linkage Algorithms Using Complete Linkage Clustering

**DOI:** 10.1371/journal.pone.0154446

**Published:** 2016-04-28

**Authors:** Abdullah-Al Mamun, Robert Aseltine, Sanguthevar Rajasekaran

**Affiliations:** 1 Department of Computer Science and Engineering, University of Connecticut, Storrs, Connecticut, United States of America; 2 Institute for Public Health Research, University of Connecticut, East Hartford, Connecticut, United States of America; Qom University, ISLAMIC REPUBLIC OF IRAN

## Abstract

Data from different agencies share data of the same individuals. Linking these datasets to identify all the records belonging to the same individuals is a crucial and challenging problem, especially given the large volumes of data. A large number of available algorithms for record linkage are prone to either time inefficiency or low-accuracy in finding matches and non-matches among the records. In this paper we propose efficient as well as reliable sequential and parallel algorithms for the record linkage problem employing hierarchical clustering methods. We employ complete linkage hierarchical clustering algorithms to address this problem. In addition to hierarchical clustering, we also use two other techniques: elimination of duplicate records and blocking. Our algorithms use sorting as a sub-routine to identify identical copies of records. We have tested our algorithms on datasets with millions of synthetic records. Experimental results show that our algorithms achieve nearly 100% accuracy. Parallel implementations achieve almost linear speedups. Time complexities of these algorithms do not exceed those of previous best-known algorithms. Our proposed algorithms outperform previous best-known algorithms in terms of accuracy consuming reasonable run times.

## Introduction

Health agencies keep track of patients′ health information and at the same time records of a patient reside in multiple data sources. All the records of a patient may be needed to accurately diagnose a disease or prescribe medicine for a disease for the patient [1, 2]. Disease evolution, drug discovery and side effects of a drug may require analysis of health records across these data sources [3, 4]. Record linkage, for example, can be used to merge records across educational databases, employment history, and family evolution to analyze an individual’s characteristics. It has also applications in similarity detection in digital documents [5, 6], master data management [7], social networking [8], historical research [9], gene expression [10–13], information science [14], health psychology [15], data mining [16, 17], etc.

Record linkage [18] integrates records across multiple data sources as well as identifies records pertaining to same individuals. Now-a-days millions of records are stored and maintained in data sources electronically. Connections among these records provide better understanding of relationships of these data sources. Exact same records exist in multiple databases. Sometimes records get polluted unintentionally due to typing error, similarity in pronunciation, etc. All of these issues make the record linkage problem very challenging and critical. Efficient algorithms are inevitable to address this problem.

Fortunately, a large number of algorithms are available in the real world [19]. A naïve algorithm compares each pair of records to find matches. It may produce expected results but has a high time complexity. Therefore algorithms have been devised to provide best possible results within a manageable time. We have previously proposed single linkage hierarchical clustering based solutions [20] for this record linkage problem. These algorithms provide very fast solutions in finding clusters of individuals with a high accuracy.

In this paper we propose a complete linkage hierarchical clustering based solution for this problem. Single linkage solution works fine for real life applications. But it has a chaining problem. We discuss the problem elaborately in this paper. Our newly devised algorithms not only solve this chaining problem but also assure expected output. We also develop an efficient parallel version of this algorithm. Our experimental results substantiate our claim.

## Background and Significance

Record linkage [21, 22] identifies record matches across different data sets even if they have no universal identifier. The problem is to group similar records so that each group contains all records of one individual only. This problem is no more than trivial if the records do not get contaminated. Often errors are introduced unintentionally while typing, due to sound similarity, etc. Every group of similar records can be thought of as a cluster. Every cluster should contain only the records of a single person and it should contain all the records of this person. Several types of clustering algorithms such as *k*-means clustering, fuzzy clustering, hierarchical clustering, graph-based clustering, etc. are widely available [23]. Our proposed algorithms are based on hierarchical clustering [24]. This requires linkage criteria that define how distances are measured between any two clusters. Single linkage and complete linkage clustering are popular in use. In single linkage, the distance between two clusters *A* and *B* is computed as the minimum distance between a point (i.e., a record) in *A* and a point in *B*. In complete linkage, the distance between two clusters *A* and *B* is computed as the maximum distance between a point in *A* and a point in *B*. Therefore single linkage clustering can be thought of as the nearest neighbor clustering and complete linkage clustering can be thought of as the farthest neighbor clustering. In addition to defining the distance between two clusters, we also have to define the distance between two records. There are many distance measures that can be used for the records. Edit distance or Levenshtein distance calculates the number of insertions, deletions and substitutions required to transform one string to the other. (We can think of every record as a string of characters). Manhattan distance computes only the number of mismatches. There exist some other distance calculation methods such as Euclidean distance, maximum distance, etc. We have used complete linkage hierarchical clustering for our algorithms. These algorithms generally use edit distance, reversal edit distance and truncation edit distance calculation methods although our algorithms can support any distance measure. Reversal edit distance and truncation edit distance also use edit distance calculation methods.

## Related Works

A naïve or brute force algorithm compares every pair of records and hence takes too much time. There exist a large number of efficient algorithms [25, 26]. [27, 28] define data cleansing and record linkage. They also present a literature survey for many proposed or developed methodologies for entity resolution and record linkage. A relational clustering algorithm uses both attribute and relational information to integrate entities [29]. Discussions about deduplication quality and data linkage measurement involve different linkage processes and issues [30]. Limitations in record linkage algorithms have also been discussed in the literature [31]. The EMH algorithm (based on expectation maximization) provides better decision rules employing probability estimates [32]. There exist some other probabilistic methods for record linkage problems [33, 34]. A hybrid Markov chain Monte Carlo algorithm calculates transitive linkage probabilities across records and uses this information for post-processing procedures such as logistic regression [35]. Relational probability model can solve the citation-matching problem [36]. Records across multiple data sets may contain variations as well as errors [37]. Edit distance calculation has been used widely to compute variations between records [38]. Case patient algorithm includes ‘Jaro—Winkler’, ‘Soundex’ and ‘weight matching’ for distance computation [39]. Record linkage has also applications in record matching [40], text correction [41], substring matching [42], etc. Relational dependencies among different fields improve record linkage processes by reducing errors [43, 44]. Conditional models for record linkage problem can handle varieties of features of input data sets independent of their dependencies [45, 46].

Blocking and indexing have been used extensively for faster computation by removing many unnecessary pair comparisons [47–49]. Traditional blocking, sorted neighborhood indexing, *Q*-gram-based indexing, suffix array-based indexing, canopy clustering, and string-map-based indexing are popular blocking techniques for reducing comparison space. [50] proposes Q-gram fingerprinting as a blocking technique. It transforms records into bit vectors and filters pairs of bit vectors using multibit trees. FEBRL [51, 52], FRIL [53, 54], Intelliclean [55] are well-known and widely used record linkage algorithms and tools. FEBRL uses three different indexing methods namely standard blocking method, sorted neighbourhood approach, and *n*-grams. It has a parallel implementation using MPI with python. FRIL is another good tool for record linkage with many options. It employs nested loop join (NLJ) and the sorted neighborhood method as search methods. Hierarchical clustering based solution has been popular for record linkage [56–59]. Given the exponential growth in data sizes, parallel solutions are inevitable [57–63]. Some efficient data integration algorithms have shown very high accuracies [64, 65]. Recently developed single linkage hierarchical clustering algorithms outperform these algorithms [20]. In this paper we propose sequential and parallel record linkage algorithms that use complete linkage clustering. These algorithms offer improved accuracies and have the potential of having a greater impact on real world applications.

## Methods

We propose sequential and parallel record linkage algorithms, which use complete linkage hierarchical clustering. These algorithms employ single linkage algorithms [20] as a preprocessing step to generate intermediate clusters. Complete linkage method is applied within each of these clusters. We employ some post processing steps to fine-tune the clusters thus generated.

### Sequential Algorithm

**Fig 1:**
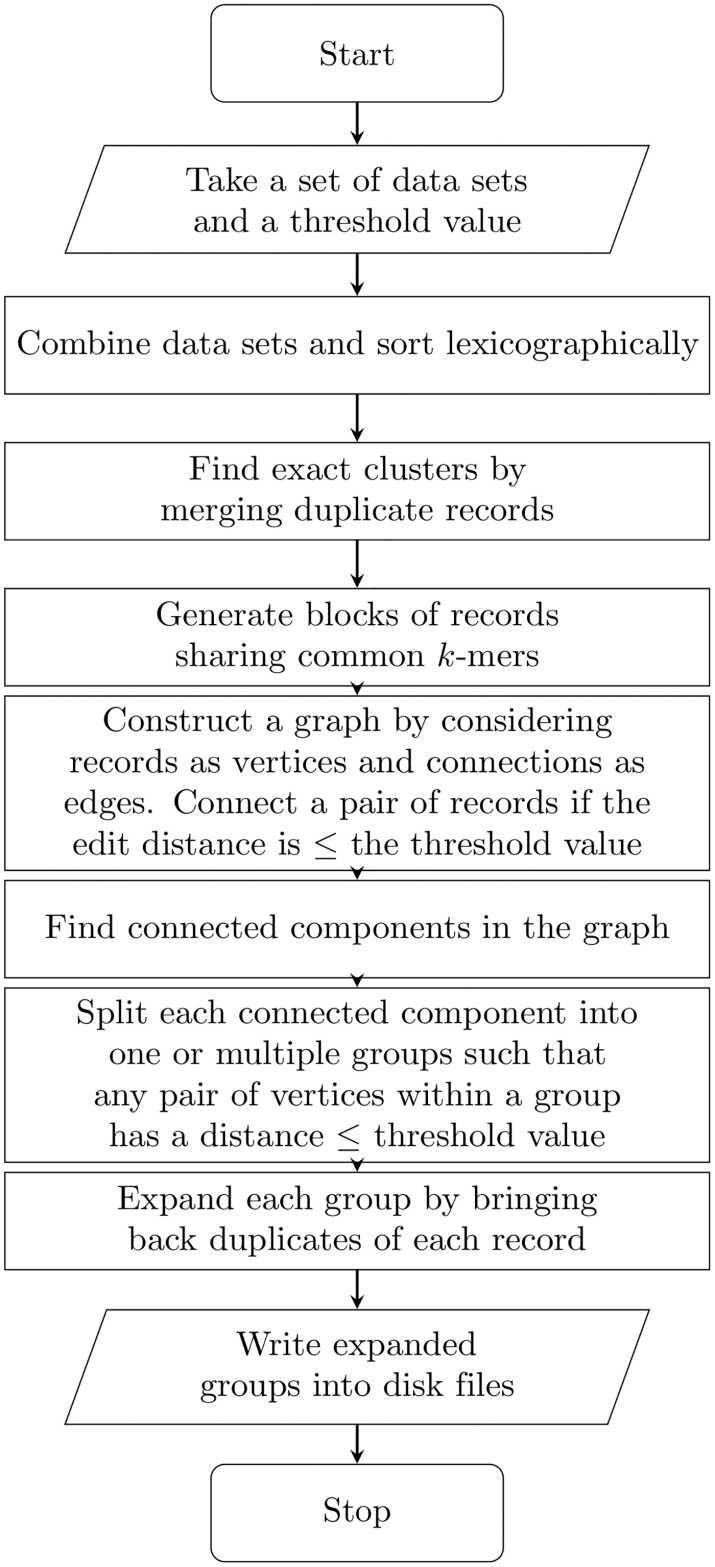
A flow chart describing all steps involved in RLA-CL.

RLA-CL (Record Linkage Algorithm—Complete Linkage) works in several phases and each of these phases consists of possibly multiple steps. Steps involved in RLA-CL are blackshown in Fig 1.

RLA-CL first sorts the records and identifies duplications. As different data sets may have different numbers and types of attributes, it takes pairs of data sets in which one of them has a subset attribute types of the other. Then the algorithm sorts them using efficient radix sort on common attributes. Exact matches will be adjacent in the sorted array. We do this sorting for each pair of data sets meeting our required criteria. We accumulate all of them and eliminate duplicates by merging them into the same clusters. This single phase removes many records from further consideration and shrinks the data sets. A simple example may simplify the working process of this phase. Let *A*, *B*, and *C* be three input data sets. *A* has *a*, *b*, *c*, and *d* as attribute fields, *B* has *a* and *d* and *C* has *a*, *d*, and *e*. Note that the attributes in *B* form a subset of the attributes in *A*. Also, the attributes in *B* form a subset of the attributes in *C*. We sort *A* and *B* together; we also sort *B* and *C* together. The attributes in *A* form neither a subset nor a superset of the attributes in *C*. Thus we do not sort *A* and *C* together. After sorting the records of *A* and *B* data sets, we accumulate duplicate records into clusters. We do the same process for *B* and *C* data sets. Then the algorithm merges these two arrays of clusters obtained from merging *A* with *B* and *B* with *C* data sets. This exact matching phase identifies all possible duplicates and unifies them into clusters. Therefore the remaining phases of the algorithm have to handle these reduced data sets only, which form a subset of the initial data sets. We have shown this phase in Algorithm 1.

**Algorithm 1** Find Exact Clusters

**Input**: A list of records

**Output**: A set of clusters of identical records

1: **procedure** FindExactCluster

2:  **for** each pair data set {*X*, *Y*} **do**

3:   **if** *attributes*_*X*_ ⊆ *attributes*_*Y*_ **then**

4:    Combine records from *X* and *Y*;

5:    Sort lexicographically using radix sort;

6:    Merge duplicate records by creating clusters of identical records;

7:    Remove duplicate records;

8:   **end if**

9:  **end for**

10:  Merge clusters generated from all pairs;

11:  **return** the set of exact matched clusters.

12: **end procedure**

Exact matching results in clusters of records. From each cluster we pick only one representative for further processing. In this way we make our algorithms independent of the number of input data sets and they can identify similar records within a data set as well as across different data sets.

Comparison between every pair of records is time consuming and impractical. Blocking helps to reduce the number of pairs to be compared. We employ *k*-mers or *k*-substrings of an attribute for blocking. If the blocking attribute contains only English letters, numbers, or alphanumeric values, then we consider only 26^*k*^, 10^*k*^, or 36^*k*^ blocks, respectively. Each block has only those records having at least one *k*-mer of the blocking field in common. If *l* is the length of the attribute of a record, then this record goes to (*l* − *k* + 1) blocks. If two records belong to the same person and if an attribute slightly differs in these two records, then there is a good chance that the two attribute instances will still have a common *k*-mer and hence the two records will fall into at least one block together. We measure distances among records in each block. We generally employ edit distance, reversal distance and truncation distance calculation methods although every suitable distance calculation works perfectly with our algorithm. Edit distance calculates the minimum number of insertions, deletions and substitutions of characters needed to change one string to the desired one. If *S*_1_ = “algrilhmss” and *S*_2_ = “algorithms”, then we can convert *S*_1_ to *S*_2_ using the following operations: insert ‘o’ at index 3 of *S*_1_, replace ‘l’ to ‘t’ at index 5 and delete ‘s’ from index 9 of *S*_1_. This algorithm discards many calculations by checking when the distance surpasses the user-defined threshold value. Therefore we have to choose a suitable threshold value dependent on our input data accuracy. Threshold value defines the maximum number of errors allowed in the input records. For the above example if the threshold value is not less than 3, then the algorithm integrates them into a single cluster. These steps have been shown in Algorithm 2.

**Algorithm 2** Perform Single Linkage Clustering

**Input**: A set of exact matched clusters and a threshold value

**Output**: A set of single linkage clusters

1: **procedure** ComputeSingleLinkageCluster

2:  Take a record from every exact matched cluster as a representative;

3:  In the next steps by a record we mean a representative record;

4:  **for** each attribute in a user defined attribute list **do**

5:   Create blocks of records sharing the same *k*-mer;

6:   **for** each block **do**

7:    Consider a graph where records are vertices and

8:    connections among them are edges;

9:    Connect two vertices if the distance (edit distance is one of the distance

10:    calculation methods) between them is at most the user defined

11:    threshold value;

12:   **end for**

13:  **end for**

14:  Remove multi-edges and self loops to make the graph simple;

15:  Find connected components of this graph;

16:  **return** the set of connected components in the above graph.

17: **end procedure**

If records are considered as vertices and distances not above threshold value as edges, then we get an undirected graph. We remove multi-edges between pairs of vertices and self-loops to convert it into a simple graph. We find all the connected components of the graph. These connected components are intermediate clusters generated by single linkage clustering method. This is the third phase of our algorithm.

Our next phases work on only records within each cluster. Every cluster typically contains a small number of records integrated by single linkage clustering. Single linkage clustering often traps in a chaining problem. Let *A*, *B*, and *C* be records, where *A* = “sweat, exercise, gymnesium” having status, type, and place as attributes, *B* = “sheat, gymnesium” with status and place as attributes and *C* = “heat” having status as the attribute. Let the threshold value be 1. Therefore *A* and *B* are in one cluster, and *B* and *C* are in another cluster, but the distance between *A* and *C* is 2, which is above the threshold value. According to our first three phases all the three records should be considered in the same cluster. Complete linkage removes this problem. It may merge *A*, *B* in a cluster and *B*, *C* in another cluster or *A*, *B* in a cluster and *C* in another cluster, and so on. It never merges *A*, *B* and *C* in a single cluster.

The fourth phase starts with considering every record in a cluster as a cluster having only one record. Then the algorithm measures distances among each pair of clusters and populating them in 2-*d* matrices. From these distances we generate a vector having minimum distances from every single record cluster. The algorithm finds the minimum of them, and if this minimum distance is not above the threshold value, then it merges these two clusters into one cluster and updates the distance matrix and vector. When we calculate the minimum distance for a cluster, we measure distances of the furthest elements between every pair of clusters having this cluster at one side and take the minimum of them. This process continues till the minimum distance does not surpass the threshold value. We eventually get clusters of records of individuals using complete linkage clustering.

**Algorithm 3** RLA-CL (Record Linkage Algorithm using Complete Linkage Clustering)

**Input**: A set of data sets and a configuration file

**Output**: A set of complete linkage clusters

 **procedure** RLA-CL

  Find exact clusters using **Algorithm 1**;

  Compute single linkage clusters by **Algorithm 2**;

  **for** each single linkage cluster **do**

   Consider every record of the cluster as a single node cluster;

   Generate a 2 − *d* square matrix where each entry contains the minimum distance

   between pairs of clusters; Each row of the matrix corresponds to a cluster;

   Generate a vector of minimum distances for each cluster;

   **while** the matrix has more than 1 row **do**

    Merge clusters if the minimum distance between them is no more than the

    user defined threshold value;

    Update the matrix and vector;

   **end while**

   Check whether merging is possible among the generated clusters;

   Use a priority list to resolve ambiguity in finding a perfect cluster

   for each record;

  **end for**

  Merge these clusters with records from exact matched clusters;

  **return** these complete linkage clusters.

 **end procedure**

The fourth phase easily eliminates the problem of merging all the records in a single cluster generated by the chaining phenomenon. But which cluster should contain which records is now a challenging task. We employ a post-processing phase to fine-tune the generated complete-linkage clusters. We require a user-defined priority list of attributes to complete this phase. We assign each priority attribute a score. We take one record from one cluster and check in which cluster it matches the best. The error-free matching with higher priority attributes, clusters having the highest number of priority attributes, etc. determine the destination cluster. This process meets the user-expectations astonishingly in real world applications. Algorithm 3 describes every step of the algorithm.

We can explain the above algorithm using a simple example. Data set *A* has 3 records {“Cade”, “Bale”, 05011976}, {“Cade”, “Bolt”, 05021986}, and {“Thor”, “Glenn”, 12011990}, and data set *B* has 2 records namely {“Thor”, “Glenn”, 12011990} and {“Cade”, “Balt”, 05011976}. Both of these data sets have first name, last name and date of birth attributes. Let the blocking field be first name; comparing attributes be the first name and last name; the priority field be date of birth; and the threshold value be 1. RLA-CL first accumulates these five records and sorts. It finds four exact matched clusters. Only one cluster {{“Thor”, “Glenn”, 12011990}, {“Thor”, “Glenn”, 12011990}} has two records having the same first name and last name. Then the algorithm creates blocks on the first name for all of the four representative records. After blocking and constructing linkages, we find 2 clusters. One is {{“Thor”, “Glenn”, 12011990}} and the other is {{“Cade”, “Bale”, 05011976}, {“Cade”, “Bolt”, 05021986}, {“Cade”, “Balt”, 05011976}}. The post processing phase finds an inconsistency: the “Balt” record may go with the “Bolt” record or the “Bale” record since the edit distance value in both cases is 1 and the threshold value is 1. To break this tie, the priority field date of birth helps us to combine the “Balt” record with the “Bale” record. Aftre expanding exact matched records we get 3 clusters {{“Thor”, “Glenn”, 12011990}, {“Thor”, “Glenn”, 12011990}}, {{“Cade”, “Bale”, 05011976}, {“Cade”, “Balt”, 05011976}}, and {{“Cade”, “Bolt”, 05021986}}.

### Analysis

We analyze the time complexity by aggregating time complexities of all the steps. Step 1 calls radix sort for at most *D*^2^/2 data sets, where *D* is the number of data sets. If *D* = 10, which is very high for real world applications, the sorting algorithm is called at most 50 times. As radix sort is a linear time algorithm, this step consumes a linear amount of time on the number of records contained in those pairs of data sets. Step 1 reduces the number of records significantly in practical applications. Let the initial number of records be *N* and this reduced number be *N*′. *K*-mer blocking is typically done on alphabet, number or alphanumeric values which generates 26^*k*^, 10^*k*^ or 36^*k*^ blocks, respectively. If a record length is *l*, then it should be in (*l* − *k* + 1) blocks. To calculate blocking information of all the records, step 2 takes at most (*l*′ − *k* + 1)*N*′ time, where *l*′ is the maximum length of any blocking attribute. Step 3 is the most time consuming step as it measures distances between records in every block. Let *b* be the number of blocks, *b*_*n*_ the average number of records in these blocks and *L* be the maximum aggregated length of common attributes of records. Then this step takes $O(b{b_{n}}^{2}L\tau )$ time, which can be written as $O(b_{n}N^{\prime }L\tau )$ as $bb_{n}=O\left(N^{\prime }\right)$. Step 4 scans through the generated graph and finds connected components. This step takes linear time in the number of records and connections, which is $O(N^{\prime })$. Steps 6 and 7 work on individual clusters that contain small numbers of records. If the number of these clusters is *C* and each cluster may contain O(D) records, then these steps take $O(D^{2}C)$ time that may be thought of as $O(DN^{\prime })$, where $DC=O(N^{\prime })$. We see that step 3 dominates the running time. Overall the running time is $O(b_{n}N^{\prime }L\tau )$, where *b*_*n*_ is the average number of records in a block (in step 3), *N*′ is the number of clusters by exact matching, *L* is the maximum aggregated length of the common attributes of records and *τ* is the user-defined threshold value.

### Parallel Algorithm

We observe that the above RLA-CL algorithm has several phases, and almost all of these phases have independent working processes. For example, the distance calculation is done within each block. Therefore processors can perform linkage calculations independent of the others. Some steps are difficult to be parallelized optimally. For them we provide experimentally optimized solutions. Some steps are trivial to parallelize. Here we propose the PRLA-CL (Parallel Record Linkage Algorithm—Complete linkage) algorithm. One processor handles the input, output and collaboration with the other processors and is called the master processor and all the other processors are referred to as slave processors.

**Algorithm 4** PRLA-CL Parallel Record Linkage Algorithm using Complete Linkage Clustering

**Input**: A set of data sets and a configuration file

**Output**: A set of complete linkage clusters

1: **procedure** PRLA-CL

2:  The Master reads data from the input files;

3:  The Master broadcasts data;

4:  **for** each processor **do**

5:   Determine which pairs of data sets should be sorted;

6:   Remove duplicates and merge records;

7:  **end for**

8:  The Master collects and merges all exact matched clusters;

9:  The Master distributes nearly uniformly representative records to each processor;

10:  **for** each processor **do**

11:   Create blocks of records sharing the same *k*-mers;

12:  **end for**

13:  The Master collects and merges all blocking information;

14:  The Master distributes block lists to all the processors nearly uniformly;

15:  **for** each processor **do**

16:   **for** each block in block list **do**

17:    Construct a graph where the records are vertices and the connections

18:    among them are edges;

19:    Connect two vertices if the distance (edit distance is one of the

20:    distance calculation methods) between them is at most the

21:    user defined threshold value;

22:   **end for**

23:  **end for**

24:  The Master accumulates edge lists from each processor;

25:  The Master finds connected components using these lists just as we do

26:  in **Algorithm 3**;

27:  The Master distributes clusters data uniformly to all the processors;

28:  **for** each processor **do**

29:   Perform complete linkage clustering and post processing

30:   (same as in **Algorithm 3**);

31:  **end for**

32:  The Master collects these clusters;

33:  The Master merges these clusters with records from initial exact matching clusters;

34:  **return** these complete linkage clusters.

35: **end procedure**

As displayed in Algorithm 4, after receiving data from the master, every processor selects pairs of data sets such that attributes of one data set cover all the attributes of the other data set. Then we accumulate records from each pair. Every processor sorts a specific range of records lexicographically. This range is chosen according to a prefix value of concatenated attributes of each record. If we choose the first 2 characters from each record, there are 676 combinations. If we have *p* processors, then every processor can keep track of records starting with $\frac{676}{p}$ character combinations. The master collects and merges all the exact match records. Then the master chooses a representative from every exact matched group. Then it sends nearly an equal number of records to each of the slave processors. The slave processors generate blocks of records sharing some common *k*-mers. The master collects this blocking information. It then sorts blocks according to the number of records they contain. Then the master groups some blocks and aggregates squares of the numbers of all records in that group. The master does this grouping in such a way that all the groups have almost the same aggregate value. Then each processor finds the edge lists. The master collects them and finds connected components. Then the master splits these connected components equally among all the other processors. All of them compute the complete linkage clusters within each component. The master gathers all the clusters and expands every representative record by all of its exact matched records.

### Analysis

This parallel algorithm distributes most of the work uniformly across all the processors. Major portions of them have been performed independently. Therefore communication cost is negligible with respect to the computational cost. Some steps have to be explained elaborately.

Step 1 takes O(N) time to read *N* records from *D* data sets and broadcast them. We see from the sequential algorithm that some pairs of data sets should be sorted to find duplicate records. In PRLA-CL, every processor determines those pairs of data sets. To compare among records, we concatenate common attributes of those records. We take the first 3 characters from each concatenated string. There may be *s* = 26^3^, 10^3^ or 36^3^ divisions of records if the characters are from English alphabet, number or alphanumeric values, respectively. Every processor sorts *s*/*p* divisions and removes duplicates by generating exact matching clusters. Although each processor does not get the same amount of records, the overall task is almost the same and the consumed time is really negligible compared to the other computations. Experimental results verify this statement. Therefore, if *s*_*n*_ is the maximum number of records of one division, then step 2 takes $O(ss_{n}/p)$ time which is O(N/p), where $ss_{n}=O\left(N\right)$. In step 3 every processor performs blocking on *N*′/*p* records which uses $O(N^{\prime }\left(L-l+1\right)/p)$ time. In [20] we see some efficient techniques to distribute blocks among the processors. Step 4 consumes $O(b_{p}{b_{pn}}^{2}L\tau )$ time, where *b*_*p*_ is the average number of blocks in a processor and *b*_*pn*_ is the average number of records in a block. Step 5 is straightforward as the master handles the collected data and finds the connected components in linear time in *N*′ and number of connections, which is $O(N^{\prime })$. In step 6 the master distributes clusters in the same way it did for blocking in step 4. Every processor gets almost the same amount of workload to find complete linkages among the records. We assume that *C* is the number of intermediate clusters. Therefore, each processor does work in $O(D^{2}C/p)$ or $O(DN^{\prime }/p)$ time. We see that the parallel algorithm has been perfectly parallelized. Experimental results show almost linear speed-up.

## Results

We have implemented RLA-CL in C++ and PRLA-CL in C++ with MPI library. We deployed them on a HPC cluster having processors of 12 Intel Xeon X5650 Westmere cores and 48 GB RAM.

FEBRL [51, 52] is a popular record linkage system. It generates clusters of very high accuracy. TPA(FCED) [64] achieved a similar accuracy with much less time. From Table 6 of [64] we see that TPA(FCED) took 203 ms in an experiment, whereas FEBRL needed 1284 ms. We outperformed TPA(FCED) by devising a novel RLA algorithm [20]. The implementation attained the same accuracy while being several times faster. The RLA paper integrated and analyzed some experimental results on real and simulated data sets. Those results exposed its efficiency and accuracy in real as well as simulated data sets. Those real data sets contained a very low percentage of errors. RLA algorithm works really fine on real data sets. But yet we see it achieved not more than 98% accuracy for real data sets. Accuracy on simulated data sets varies widely due to a broad ranges of errors. In our experiments we count the possible traps of TPA(FCED) and RLA algorithms and show how RLA-CL finds the expected output. We will also show how blocking information affects its performance. We will evaluate efficiency of record linkage algorithm using complete linkage hierarchical clustering over single linkage clustering. We have employed only simulated data sets, which contain much more errors than normal, to verify our statements of efficiency and accuracy of RLA-CL.

### Generation of Simulated Data Sets

We generated three types of synthetic data sets. The first type has a data set of 1 million records. We made 10 copies of this data set. Then we introduced one insertion, deletion, or substitution error in the last name attribute of every record with a 15% probability. This means that around 15% of all the records in a data set have one mismatch from its original record. These data sets have the first name and SSN attributes along with some other attributes. We have taken an equal number of records from each data set in our experiments. If the number of records is 1 million, every data set contributes 100,000 records. SSN is a unique attribute for every record. We compute the accuracy using this attribute. This type is used to compare performances among TPA(FCED) [64], RLA [20] and RLA-CL implementations. The second type of data sets were generated from the previous 1 million records. We copied this data set two more times. Then we inserted, deleted, or substituted one symbol in the last name of each record. This means that every record has at least one mismatch from its original record. We used four original data sets, and these two data sets three times. The third type is used for analysing different aspects of RLA-CL. The original data set has 1,600,000 records. We generate three copies of this data set. We remove different attributes from each data set. Then we introduce one insertion, deletion, or substitution error in the last name of every record. We analyze how RLA-CL works for different numbers and types of attributes. We have then cloned all of these three data sets.

### Sequential Algorithm

We have categorized our experimental results into three sections. The first section shows that RLA-CL outperforms RLA and TPA(FCED) in terms of accuracy and removes the chaining phenomenon. The number of blocks and types of blocking fields affect the running time and accuracy of RLA-CL. We explain them in the second section. In the third section we distribute the running time of RLA-CL and show that it does not take much time than RLA, the best-known algorithm in this category. We have divided the output data into four categories to measure accuracy. Type I includes perfect clusters. Each cluster contains all the records of an individual and does not contain any record from other individuals. Every cluster of Type II has records of only one individual, but does not include all of them. All the records of an individual mixed with some records of the other individuals are included in Type III category. A Type IV cluster has some records from one individual mixed with some records of the other individuals. Here we see that Type I clusters are the most preferred. A Type IV cluster is a truly incorrect cluster. Therefore we prefer more records in Type I category and less records in Type IV category.

Table 1 compares our newly devised RLA-CL algorithm with the previously best-known RLA algorithm as well as TPA(FCED). Number of records ranges from 100 thousands to 1 million across the five data sets. We have used the first name as the blocking field and Social Security Number as the accuracy testing attribute. We have used the edit distance calculation method on the first name and last name attributes. We have set 2 as the threshold value and 3 as the value of *k*.

**Table 1:** A comparison among TPA(FCED), RLA and RLA-CL on simulated data sets (generated with a low error rate).

No Of Records	Algorithm	Time	Record Category(%)			
			Type I	Type II	Type III	Type IV
100,000	TPA(FCED)	31.01	97.38	0.00	2.62	0.00
	RLA	2.15	97.38	0.00	2.62	0.00
	RLA-CL	3.08	99.88	0.01	0.11	0.00
200,000	TPA(FCED)	122.77	93.76	2.86	3.26	0.12
	RLA	7.77	93.76	2.86	3.26	0.12
	RLA-CL	10.4	99.05	0.73	0.20	0.02
400,000	TPA(FCED)	432.5	95.88	1.84	2.19	0.09
	RLA	26.56	95.88	1.84	2.19	0.09
	RLA-CL	32.15	99.45	0.44	0.10	0.01
600,000	TPA(FCED)	878	96.92	1.39	1.63	0.06
	RLA	54.50	96.92	1.39	1.63	0.06
	RLA-CL	62.54	99.61	0.32	0.07	0.00
800,000	TPA(FCED)	1503.53	97.57	1.09	1.29	0.05
	RLA	87.66	97.57	1.09	1.29	0.05
	RLA-CL	97.62	99.70	0.25	0.05	0.00
1,000,000	TPA(FCED)	2157.46	98.02	0.89	1.05	0.04
	RLA	129.54	98.02	0.89	1.05	0.04
	RLA-CL	141.17	99.76	0.20	0.04	0.00

**Fig 2:**
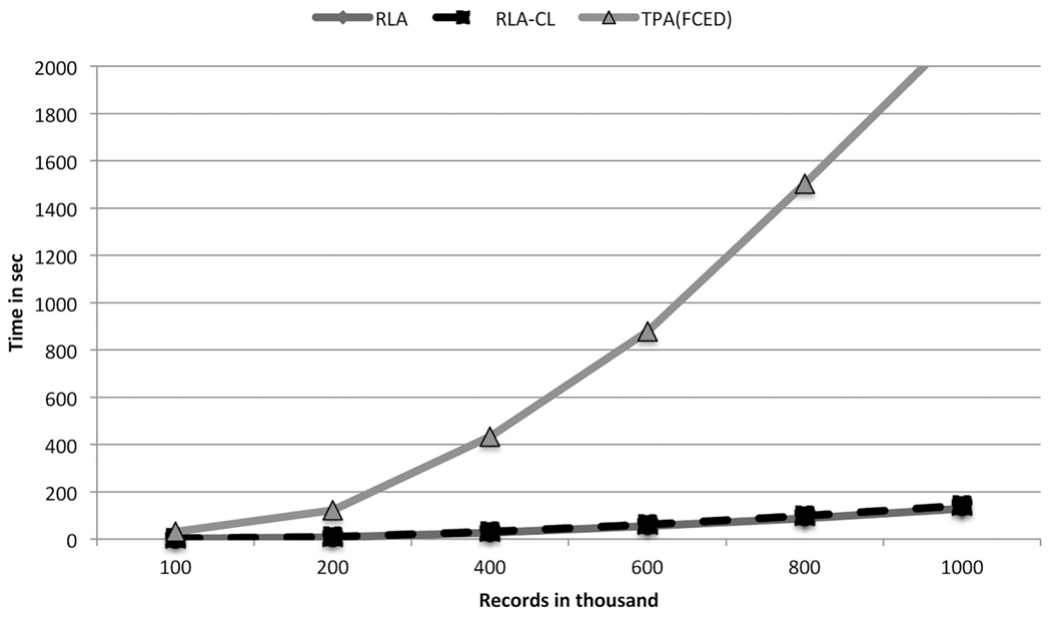
A comparison of running times of TPA(FCED), RLA and RLA-CL on simulated data sets (generated with a low error rate).

**Fig 3:**
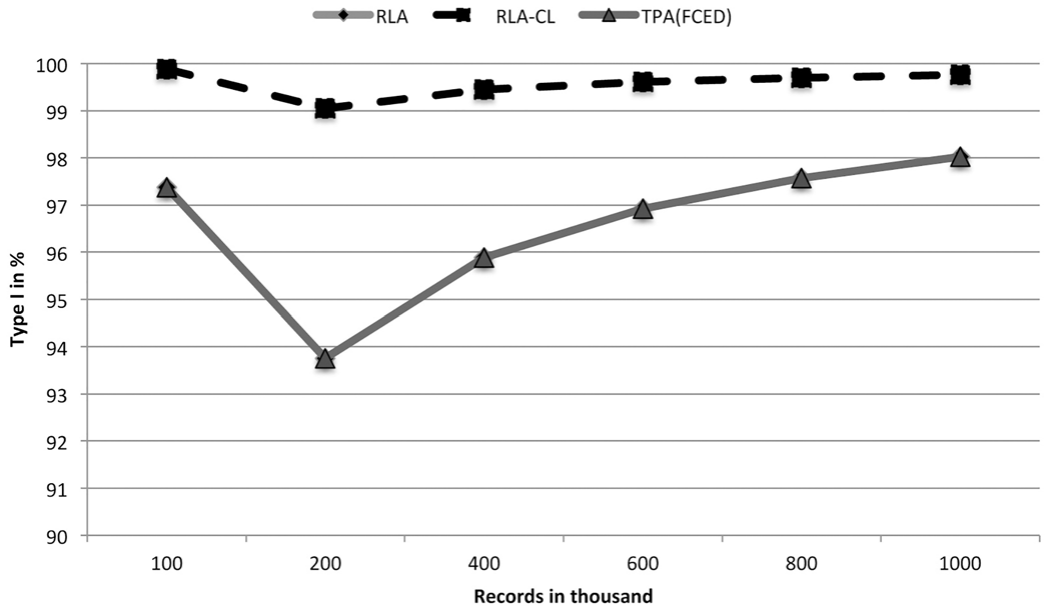
A comparison of Type I accuracies of TPA(FCED), RLA and RLA-CL on simulated data sets (generated with a low error rate).

From Table 1 we see that RLA-CL takes almost the same time as RLA. RLA and TPA(FCED) produced the same number and types of clusters. But we see TPA(FCED) takes much more time than RLA and RLA-CL. The RLA paper explained the inverse relationship between the multiplicity of exact matched records and the running time of RLA. The RLA-CL algorithm includes RLA as a preprocessing step. After preprocessing is done, the generated clusters are of small size. Therefore complete linkage among the small number of records in every cluster consumes a small amount of time. Even for 1 million records RLA-CL spends only 13 seconds more than RLA. These few seconds do complete linkage clustering and post-processing of all the single linkage clusters. Fig 2 shows this time comparison. These results show that RLA-CL provides almost 100% Type I clusters whereas RLA and TPA(FCED) produce around 96%–98% Type I clusters. If we consider 1,000,000 records of 100,000 individuals, RLA-CL only misses perfect clusters of 241 individuals whereas RLA and TPA(FCED) do not find accurately all the records of 1981 people. This difference occurs because of the chaining problem of single linkage clustering. We have shown this blackType I accuracy comparison in Fig 3.

We have seen four types of accuracy in Table 1. Accuracy can also be calculated in terms of receiver operating characteristics (ROC). For the case of two classes, ROC-based accuracy is defined as (the number of true positives + the number of true negatives)/(the total number of records). We extend this definition of accuracy to more than two classes as follows. Each cluster is associated with a user who has a majority of records in this cluster. We say that this user owns this cluster. A record in any cluster is labeled as correct if it belongs to the owner of this cluster. Now we compute the accuracy as (the number of records with correct labels)/(the total number of records). Note that this definition of accuracy is a natural extension of ROC-based accuracy to more than two classes.

**Fig 4:**
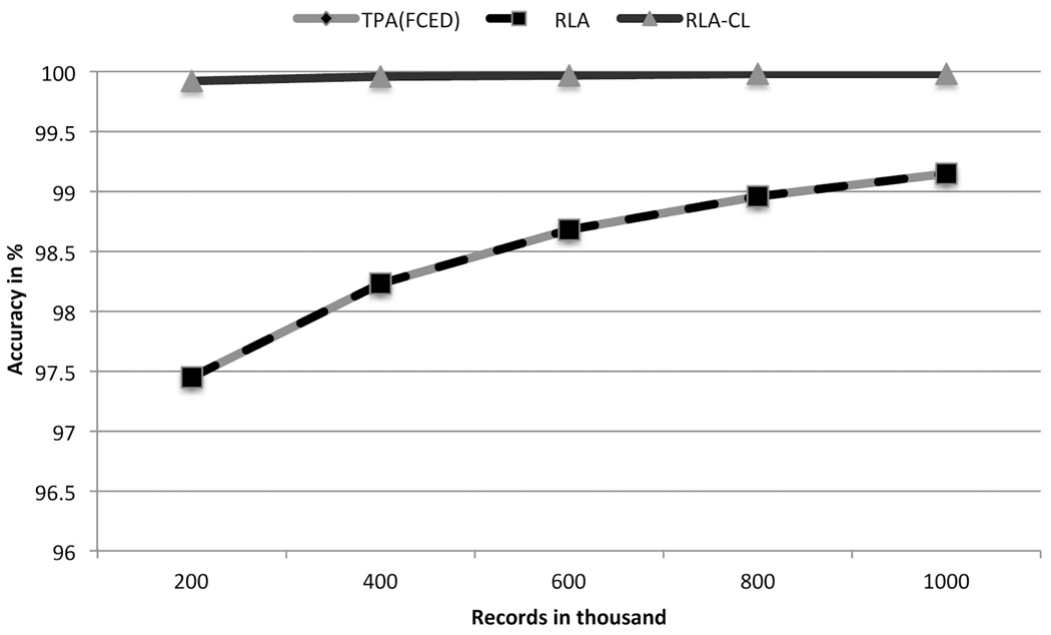
A comparison of accuracies of TPA(FCED), RLA and RLA-CL on simulated data sets (generated with a low error rate).

RLA-CL achieves more than 99.9% accuracy and TPA(FCED) and RLA achieve around 97%—99% accuracy for these data sets (shown in Table 2). Fig 4 also shows these results graphically.

**Table 2:** blackComputation of accuracy of TPA(FCED), RLA and RLA-CL on simulated data sets (generated with a low error rate).

No Of Records	Algorithm	Records With Correct Labels	Accuracy in %
100,000	TPA(FCED)	97880	97.88
	RLA	97880	97.88
	RLA-CL	99949	99.95
200,000	TPA(FCED)	194910	97.46
	RLA	194910	97.46
	RLA-CL	199836	99.92
400,000	TPA(FCED)	392930	98.23
	RLA	392930	98.23
	RLA-CL	399835	99.96
600,000	TPA(FCED)	592100	98.68
	RLA	592100	98.68
	RLA-CL	599835	99.97
800,000	TPA(FCED)	791680	98.96
	RLA	791680	98.96
	RLA-CL	799836	99.98
1,000,000	TPA(FCED)	991500	99.15
	RLA	991500	99.15
	RLA-CL	999836	99.98

We have also included results for the second type of data sets in Table 3. These data sets contain a very high error rate. Even for these data sets, RLA-CL shows almost 100% accuracy in finding perfect clusters.

**Table 3:** A comparison among TPA(FCED), RLA and RLA-CL on simulated data sets (generated with a very high error rate).

No Of Records	Algorithm	Time	Record Category(%)			
			Type I	Type II	Type III	Type IV
100,000	TPA(FCED)	31.11	96.36	0.00	3.64	0.00
	RLA	3.07	96.36	0.00	3.64	0.00
	RLA-CL	4.21	99.86	0.06	0.08	0.00
200,000	TPA(FCED)	119.62	92.49	2.89	4.58	0.04
	RLA	11.16	92.49	2.89	4.58	0.04
	RLA-CL	14.51	99.54	0.30	0.12	0.04
400,000	TPA(FCED)	421.24	94.83	1.91	3.23	0.03
	RLA	39.15	94.83	1.91	3.23	0.03
	RLA-CL	46.67	99.69	0.23	0.06	0.02
600,000	TPA(FCED)	898.64	96.07	1.46	2.44	0.03
	RLA	77.95	96.07	1.46	2.44	0.03
	RLA-CL	88.66	99.77	0.18	0.04	0.01
800,000	TPA(FCED)	1507.45	96.91	1.16	1.91	0.02
	RLA	129.79	96.91	1.16	1.91	0.02
	RLA-CL	143.19	99.82	0.15	0.03	0.00
1,000,000	TPA(FCED)	2171.43	97.47	0.95	1.56	0.02
	RLA	193.85	97.47	0.95	1.56	0.02
	RLA-CL	209.45	99.85	0.12	0.02	0.01

Sometimes one attribute may be error prone than the others. Blocking on that field produces blocks that may not hold all the records of same individuals. Multiple blocking attributes assure better results. We have explored this issue by employing three different experiments. One uses social security number (SSN) and the last name (LN) as blocking attributes, the second one uses only SSN and the last one uses only LN as the blocking attribute. We have used 5-mer on SSN, a numeric attribute, and 3-mer LN, which contains only English alphabet. Table 4 shows these comparisons in terms of running time and accuracy. We have used 6 data sets where 3 data sets have exact clone so that we could remove half of the records only after the exact matching phase.

**Table 4:** A comparison of runtime and accuracy using SSN-LN, SSN and LN as blocking fields.

No Of Records	Algorithm	Time	Record Category			
			Type I	Type II	Type III	Type IV
100,000	SSN-LN	111.51	100,000	0	0	0
	SSN	107.71	89,866	10,134	0	0
	LN	5.56	80,476	19,524	0	0
200,000	SSN-LN	252.46	199,988	4	8	0
	SSN	237.83	179,972	20,020	8	0
	LN	16.20	161,522	38,470	8	0
400,000	SSN-LN	537.83	399,952	16	32	0
	SSN	480.49	359,656	40,320	24	0
	LN	48.47	322,612	77,372	16	0
800,000	SSN-LN	1064.43	799,904	32	64	0
	SSN	822.92	719,474	80,472	48	6
	LN	169.08	644,204	155,758	32	6
1,600,000	SSN-LN	2657.35	1,599,832	56	112	0
	SSN	1912.80	1,439,608	160,290	96	6
	LN	622.12	1,290,514	309,424	48	14
3,200,000	SSN-LN	6422.64	3,199,676	96	192	36
	SSN	4261.62	2,877,986	321,824	152	38
	LN	2379.88	2,583,536	616,340	88	36

**Fig 5:**
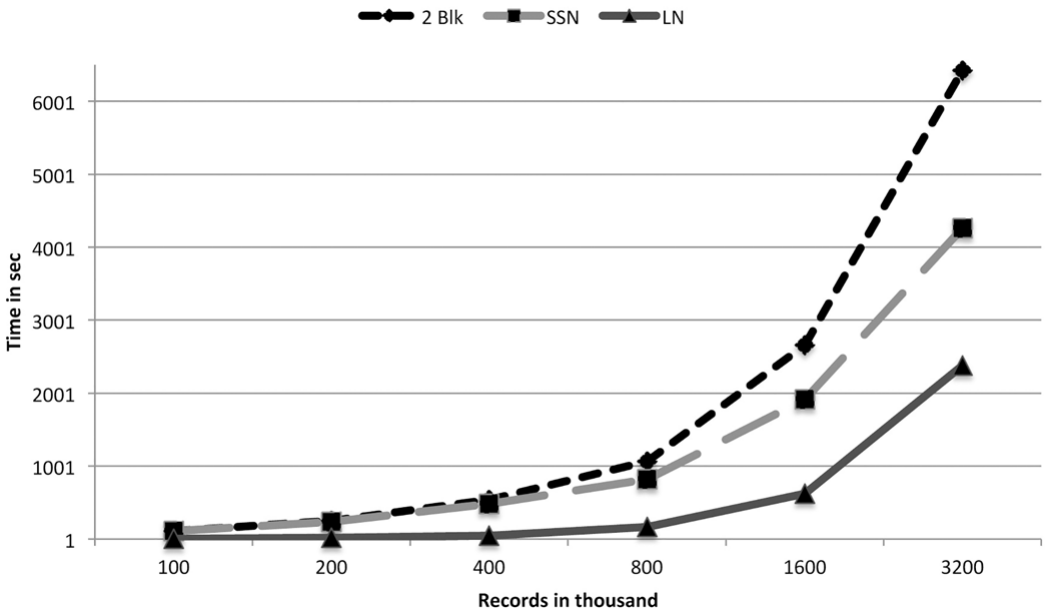
A comparison of running time for variations of blocking attributes.

**Fig 6:**
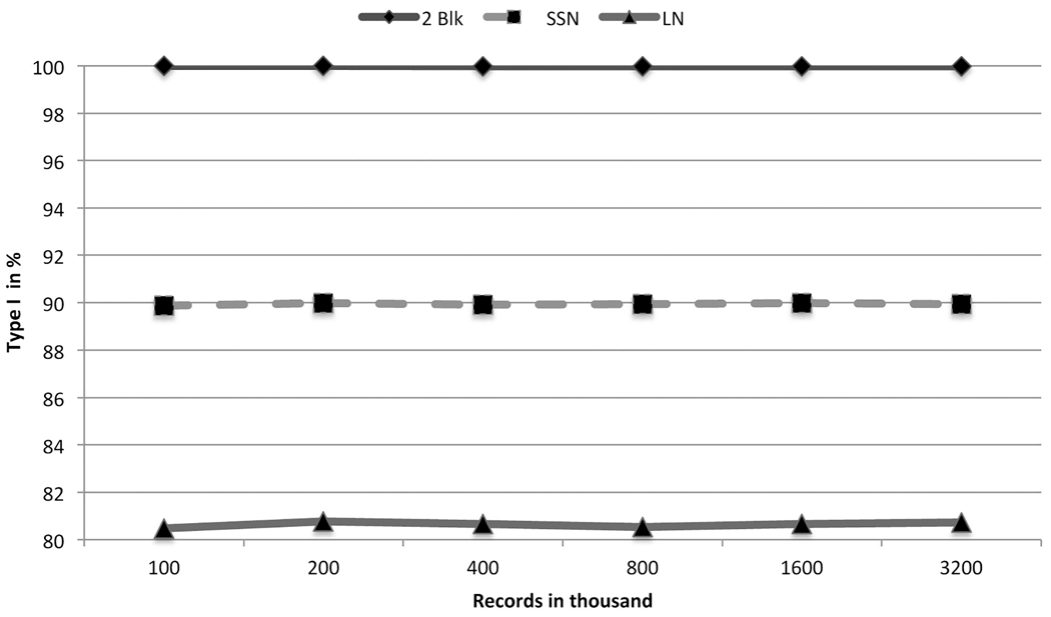
A comparison of Type I accuracy for variations of blocking attributes.

Experiments that do blocking on LN take very little time for a small number of records. But the running time increases rapidly for higher number of records. LN has an average length of 5. Therefore every record on an average goes to 3 blocks. For 100,000 records, 300,000 records are stored in 263 or 17576 blocks. Every block holds around 17 records on an average. But when we have 3,200,000 records, we have 17576 blocks to keep 9,600,000 records. Each block has to store on an average 546 records. We know that the distance calculation occurs among records densely within every block. This is the most time consuming phase of our algorithm. On the other side SSN uses 5-mers for blocking. Therefore every record goes to 5 blocks. For 100,000 records 105 or 100,000 blocks hold 500,000 records, which is 5 per block on an avearge. For 3,200,000 records this number is 160. But the most compelling reason is that some combinations of letters are more frequent than the others. This makes some blocks much larger than the others. But for numerical values every block is almost equally populated. The time needed for two attributes is the summation of these two attributes. We have depicted this scenario in Fig 5. Blocking attribute has a greater impact on accuracy. Our generated records contain errors either in the SSN or the LN. Therefore many blocks may not be able to hold records of the same individuals. But if we take blocking of two attributes, we get around 100% Type I clusters. Blocking on SSN achieves 90% and blocking on LN gets 81% Type I clusters. SSN has a better performance as each record goes to 6 blocks compared to 3 blocks for the LN attribute. Fig 6 displays the impact of blocking attributes over blackType I accuracy.

**Fig 7:**
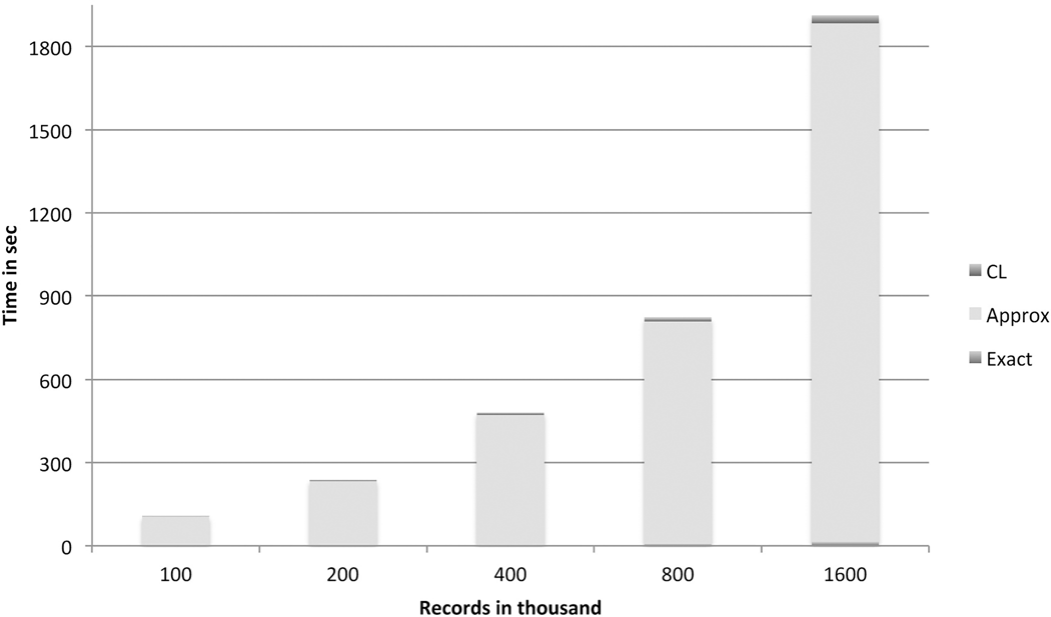
Running time distribution of RLA-CL.

Table 5 distributes the running time of RLA-CL when SSN has been used as the blocking field. Exact matching is required to remove exact duplicates. Linear time radix sorting algorithm does this step efficiently. Approximate clustering is the most time consuming phase that includes linkage calculation steps. We find clusters as connected components with almost no time. These three portions are required for both RLA and RLA-CL. The later one requires some extra time to find complete linkages. We see from the table that this value is negligible compared to approximate cluster time. We have shown these distributions in Fig 7.

**Table 5:** Distribution of running time when blocking on SSN field.

No of Records	Exact Cl T	Approx Cl T	Conn Comp T	Comp Link T	Total Time
100,000	0.77	105.34	0.01	1.59	107.71
200,000	1.90	232.25	0.03	3.65	237.83
400,000	3.62	468.84	0.06	7.97	480.49
800,000	6.36	803.65	0.08	12.83	822.92
1,600,000	13.92	1870.60	0.17	28.11	1912.80
3,200,000	29.59	4172.79	0.35	58.89	4261.62

### Parallel Algorithm

We have run our parallel algorithm on 3.2 million and 6.4 million records blocking on the last name (LN) attribute. Our parallel experiments have been tested on at most 32 cores of 4 nodes, each node having 8 cores.

**Fig 8:**
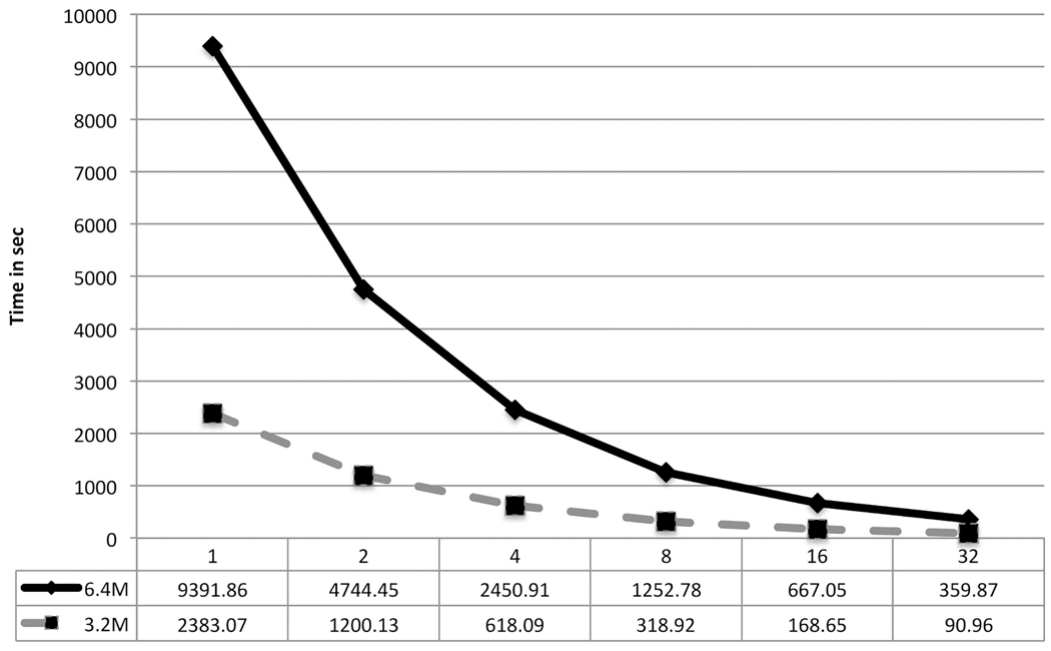
Running time of PRLA-CL for 3.2M and 6.4M records.

**Fig 9:**
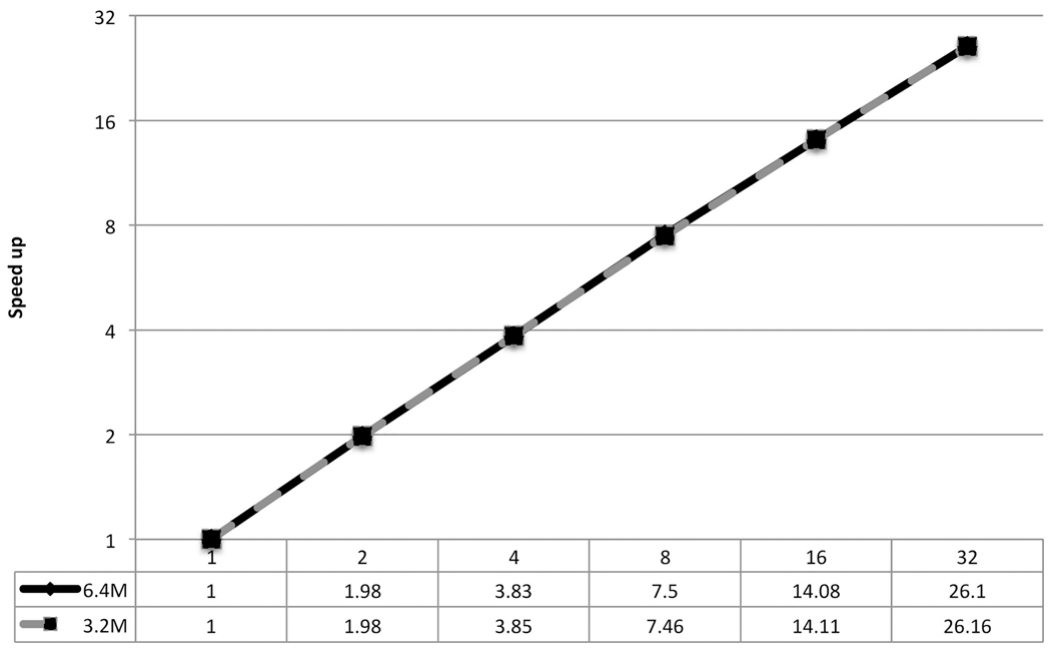
Speedup of PRLA-CL for 3.2M and 6.4M records.

Fig 8 shows the running time on different number of processors. We get almost linear speedup. These speedups have been drawn in Fig 9. We see that the most time-consuming part is the single linkage calculation among the records within individual blocks. Different blocks have different numbers of records. Even if two blocks have the same number of records, they may need different time as the time needed depends on matching of records as well as record lengths. We have distributed the runtime of PRLA-CL in Table 6. Detailed time distribution of different tasks of parallel RLA such as broadcast time, communication time, time spent by the master, blocking time, merge time, edgelist calculation time, etc. have been described in [20]. In Table 6 we have included communication time, which aggregates broadcast, communication and merge time, exact matching time, approximation clustering time that covers the generation of blocks and calculation of linkage time, finding connected components time and complete linkage time, which includes complete linkage and post-processing time. The first row shows the running time of the same data for the sequential algorithm.

**Table 6:** Distribution of running time on multiple cores.

Proc	Comm	Exact	Approx	Conn Comp	Comp Link	Total	Speedup
Seq	0	55.31	9218.79	0.68	116.19	9390.97	-
1	0	55.87	9219.32	0.69	115.98	9391.86	1.00
2	0.87	28.35	4655.38	0.63	59.22	4744.45	1.98
4	0.91	15.03	2403.09	0.65	31.23	2450.91	3.83
8	1.08	7.40	1226.59	0.70	17.01	1252.78	7.50
16	5.27	4.18	647.63	0.64	9.33	667.05	14.08
32	7.87	2.39	343.41	0.63	5.57	359.87	26.10

We have achieved 7.5 speedup for 8 cores in a single node, 14.1 for 16 cores across 2 nodes and 26.1 for 32 cores of 4 nodes. Table 6 also shows that communication time is very negligible as most of the steps of the parallel algorithm are easily parallelized. Therefore communication is needed after each phase only. These speedups are great as they are almost linear, but we can improve these speedups if we can ensure a better uniform distribution of blocks among the processors in terms of needed calculation time.

## Discussion

From the results section we see that the single linkage clustering algorithm suffers from the chaining problem. RLA-CL overcomes this problem by employing complete linkage clustering. Accuracy performance of our new algorithm sometimes depends on the number and type of blocking attributes. There is a trade-off between time spent and accuracy. Stable fields should be chosen as blocking attributes. The value of k also affects the running time and accuracy. If we use 4-mers instead of 3-mers, there will be more blocks. Each block will contain less records on an average and therefore it will cost less than before. But a 4-mer creates less substrings of records which will decrease the accuracy. RLA-CL works on different numbers and types of attributes. It does post-processing on complete-linkage clusters based on priority-list attributes. Another major factor that has a great impact on efficiency and accuracy is the threshold value. RLA applies a constant as well as a proportional threshold value and provides those results. Our new algorithm works in the same way. We can apply different threshold values on the training data sets to find out the perfect threshold value for these data sets. There is no universal threshold value for all types of data sets. Errors introduced in the data sets also have an effect on the threshold value and the performance of the algorithms. If the error rate is low, a threshold value of 1 works fine most of the time.

## Conclusions

Our newly developed record linkage algorithms using complete linkage clustering outperform previous best-known algorithms in this category. They produce more accurate results than the others. The exact matching phase sometimes shrinks much-cleaner real data sets a lot by removing duplicate records. Our experiments show that the post-processing phase generates more accurate results. Our parallel algorithm achieves almost linear speedup that can be applied over millions of records on hundreds of processors. Therefore our proposed algorithms, RLA-CL and PRLA-CL provide the best solution for record linkage problems.

## References

[1] Maizlish NA, Herrera L. A Record Linkage Protocol for a Diabetes Registry at Ethnically Diverse Community Health Centers. Journal of the American Medical Informatics Association. 2005;12(3):331–337. 10.1197/jamia.M1696 15684130 PMC1090465

[2] Victor TW, Mera RM. Record linkage of health care insurance claims. Journal of the American Medical Informatics Association. 2001;8(3):281–288. 10.1136/jamia.2001.0080281 11320072 PMC131035

[3] Clark D. Practical introduction to record linkage for injury research. Injury Prevention. 2004;10(3):186–191. 10.1136/ip.2003.004580 15178677 PMC1730090

[4] Fayyad U, Piatetsky-Shapiro G, Smyth P. From data mining to knowledge discovery in databases. AI magazine. 1996;17(3):37.

[5] Brin S, Davis J, Garcia-Molina H. Copy detection mechanisms for digital documents In: ACM SIGMOD Record. vol. 24 ACM; 1995 p. 398–409.

[6] Shivakumar N, Garcia-Molina H. Building a scalable and accurate copy detection mechanism. In: Proceedings of the first ACM international conference on Digital libraries. ACM; 1996. p. 160–168.

[7] Zhao Y, Karypis G. Evaluation of hierarchical clustering algorithms for document datasets. In: Proceedings of the eleventh international conference on Information and knowledge management. ACM; 2002. p. 515–524.

[8] Reuter T, Cimiano P, Drumond L, Buza K, Schmidt-Thieme L. Scalable Event-Based Clustering of Social Media Via Record Linkage Techniques In: ICWSM; 2011.

[9] Winkler WE, Thibaudeau Y. An application of the Fellegi-Sunter model of record linkage to the 1990 US decennial census. US Bureau of the Census. 1991;p. 1–22.

[10] Heyer LJ, Kruglyak S, Yooseph S. Exploring expression data: identification and analysis of coexpressed genes. Genome research. 1999;9(11):1106–1115. 10.1101/gr.9.11.1106 10568750 PMC310826

[11] Hawse JR, Hejtmancik JF, Huang Q, Sheets NL, Hosack DA, Lempicki RA, et al Identification and functional clustering of global gene expression differences between human age-related cataract and clear lenses. Molecular vision. 2003;9:515 14551530 PMC2831407

[12] Huang DW, Sherman BT, Lempicki RA. Systematic and integrative analysis of large gene lists using DAVID bioinformatics resources. Nature protocols. 2008;4(1):44–57. 10.1038/nprot.2008.211 19131956

[13] Dennis Jr G, Sherman BT, Hosack DA, Yang J, Gao W, Lane HC, et al DAVID: database for annotation, visualization, and integrated discovery. Genome biol. 2003;4(5):P3 10.1186/gb-2003-4-5-p3 12734009 PMC193660

[14] Vinh NX, Epps J, Bailey J. Information theoretic measures for clusterings comparison: is a correction for chance necessary? In: Proceedings of the 26th Annual International Conference on Machine Learning. ACM; 2009. p. 1073–1080.

[15] Clatworthy J, Buick D, Hankins M, Weinman J, Horne R. The use and reporting of cluster analysis in health psychology: A review. British journal of health psychology. 2005;10(3):329–358. 10.1348/135910705X25697 16238852

[16] Wong W, Liu W, Bennamoun M. Tree-traversing ant algorithm for term clustering based on featureless similarities. Data Mining and Knowledge Discovery. 2007;15(3):349–381. 10.1007/s10618-007-0073-y

[17] Ng RT, Han J. Efficient and Effective Clustering Methods for Spatial Data Mining. In: Proceedings of the 20th International Conference on Very Large Data Bases. VLDB’94. San Francisco, CA, USA: Morgan Kaufmann Publishers Inc.; 1994. p. 144–155. Available from: http://dl.acm.org/citation.cfm?id=645920.672827.

[18] Winkler WE. Matching and record linkage. Business survey methods. 1995;1:355–384.

[19] Koudas N, Sarawagi S, Srivastava D. Record linkage: similarity measures and algorithms. In: Proceedings of the 2006 ACM SIGMOD international conference on Management of data. ACM; 2006. p. 802–803.

[20] Mamun AA, Mi T, Aseltine R, Rajasekaran S. Efficient sequential and parallel algorithms for record linkage. Journal of the American Medical Informatics Association. 2014;21(2):252–262. 10.1136/amiajnl-2013-002034 24154837 PMC3932463

[21] Christen P. Data matching: concepts and techniques for record linkage, entity resolution, and duplicate detection. Springer Science & Business Media; 2012.

[22] Gu L, Baxter R, Vickers D, Rainsford C. Record linkage: Current practice and future directions. CSIRO Mathematical and Information Sciences Technical Report. 2003;3:83.

[23] Jain AK, Murty MN, Flynn PJ. Data clustering: a review. ACM computing surveys (CSUR). 1999;31(3):264–323. 10.1145/331499.331504

[24] Johnson SC. Hierarchical clustering schemes. Psychometrika. 1967;32(3):241–254. 10.1007/BF02289588 5234703

[25] Gomatam S, Carter R, Ariet M, Mitchell G. An empirical comparison of record linkage procedures. Statistics in medicine. 2002;21(10):1485–1496. 10.1002/sim.1147 12185898

[26] Winkler WE. Overview of record linkage and current research directions In: Bureau of the Census. Citeseer; 2006.

[27] Brizan DG, Tansel AU. A. Survey of Entity Resolution and Record Linkage Methodologies. Communications of the IIMA. 2015;6(3):5.

[28] Li X, Shen C. Linkage of patient records from disparate sources. Statistical methods in medical research. 2013;22(1):31–38. 10.1177/0962280211403600 21665896

[29] Bhattacharya I, Getoor L. Collective entity resolution in relational data. ACM Transactions on Knowledge Discovery from Data (TKDD). 2007;1(1):5 10.1145/1217299.1217304

[30] Christen P, Goiser K. Quality and complexity measures for data linkage and deduplication In: Quality Measures in Data Mining. Springer; 2007 p. 127–151.

[31] Winkler WE. The state of record linkage and current research problems In: Statistical Research Division, US Census Bureau. Citeseer; 1999.

[32] Winkler WE, of the Census USB, et al Improved decision rules in the fellegi-sunter model of record linkage. Citeseer; 1993.

[33] Fellegi IP, Sunter AB. A theory for record linkage. Journal of the American Statistical Association. 1969;64(328):1183–1210. 10.1080/01621459.1969.10501049

[34] Elmagarmid AK, Ipeirotis PG, Verykios VS. Duplicate record detection: A survey. Knowledge and Data Engineering, IEEE Transactions on. 2007;19(1):1–16. 10.1109/TKDE.2007.250581

[35] Steorts RC, Hall R, Fienberg SE. A Bayesian approach to graphical record linkage and de-duplication. Journal of the American Statistical Association. 2015;(just-accepted). 10.1080/01621459.2015.1105807

[36] Pasula H, Marthi B, Milch B, Russell S, Shpitser I. Identity uncertainty and citation matching In: Advances in neural information processing systems; 2002 p. 1401–1408.

[37] Christen P. A comparison of personal name matching: Techniques and practical issues. In: Data Mining Workshops, 2006. ICDM Workshops 2006. Sixth IEEE International Conference on. IEEE; 2006. p. 290–294.

[38] Levenshtein VI. Binary codes capable of correcting deletions, insertions, and reversals In: Soviet physics doklady. vol. 10; 1966 p. 707–710.

[39] Cox S, Martin R, Somaia P, Smith K. The development of a data-matching algorithm to define the’case patient’. Australian Health Review. 2013;37(1):54–59. 10.1071/AH11161 23257311

[40] Jaro MA. Advances in record-linkage methodology as applied to matching the 1985 census of Tampa, Florida. Journal of the American Statistical Association. 1989;84(406):414–420. 10.1080/01621459.1989.10478785

[41] Kukich K. Techniques for automatically correcting words in text. ACM Computing Surveys (CSUR). 1992;24(4):377–439. 10.1145/146370.146380

[42] Friedman C, Sideli R. Tolerating spelling errors during patient validation. Computers and Biomedical Research. 1992;25(5):486–509. 10.1016/0010-4809(92)90005-U 1395524

[43] Culotta A, McCallum A. Joint deduplication of multiple record types in relational data. In: Proceedings of the 14th ACM international conference on Information and knowledge management. ACM; 2005. p. 257–258.

[44] Domingos P. Multi-relational record linkage. In: In Proceedings of the KDD-2004 Workshop on Multi-Relational Data Mining. Citeseer; 2004.

[45] McCallum A, Wellner B. Conditional Models of Identity Uncertainty with Application to Noun Coreference. Advances in Neural Information Processing Systems (NIPS’04). 2004;.

[46] Lafferty J, McCallum A, Pereira FC. Conditional random fields: Probabilistic models for segmenting and labeling sequence data. In: Proceedings of the Eighteenth International Conference on Machine Learning; 2001. p. 282–289.

[47] McCallum A, Nigam K, Ungar LH. Efficient clustering of high-dimensional data sets with application to reference matching. In: Proceedings of the sixth ACM SIGKDD international conference on Knowledge discovery and data mining. ACM; 2000. p. 169–178.

[48] Steorts RC, Ventura SL, Sadinle M, Fienberg SE. A comparison of blocking methods for record linkage In: Privacy in Statistical Databases. Springer; 2014 p. 253–268.

[49] Christen P. A survey of indexing techniques for scalable record linkage and deduplication. Knowledge and Data Engineering, IEEE Transactions on. 2012;24(9):1537–1555. 10.1109/TKDE.2011.127

[50] Bachteler T, Reiher J, Schnell R. Similarity filtering with multibit trees for record linkage German Record Linkage Center, Nuremberg, Working Paper WP-GRLC-2013-02. 2013;.

[51] Christen P, Churches T, Hegland M. Febrl—a parallel open source data linkage system In: Advances in knowledge discovery and data mining. Springer; 2004 p. 638–647.

[52] Christen P. Febrl: a freely available record linkage system with a graphical user interface. In: Proceedings of the second Australasian workshop on Health data and knowledge management-Volume 80. Australian Computer Society, Inc.; 2008. p. 17–25.

[53] Jurczyk P, Lu JJ, Xiong L, Cragan JD, Correa A. FRIL: A tool for comparative record linkage. In: AMIA annual symposium proceedings. vol. 2008. American Medical Informatics Association; 2008. p. 440. PMC2656092 18998844

[54] Jurczyk P, Lu JJ, Xiong L, Cragan JD, Correa A. Fine-grained record integration and linkage tool. Birth Defects Research Part A: Clinical and Molecular Teratology. 2008;82(11):822–829. 10.1002/bdra.20521 18985680

[55] Lee ML, Ling TW, Low WL. IntelliClean: a knowledge-based intelligent data cleaner. In: Proceedings of the sixth ACM SIGKDD international conference on Knowledge discovery and data mining. ACM; 2000. p. 290–294.

[56] Rajasekaran S. Efficient parallel hierarchical clustering algorithms. IEEE Transactions on Parallel & Distributed Systems. 2005;(6):497–502. 10.1109/TPDS.2005.72

[57] Li X. Parallel algorithms for hierarchical clustering and cluster validity. Pattern Analysis and Machine Intelligence, IEEE Transactions on. 1990;12(11):1088–1092. 10.1109/34.61708

[58] Olson CF. Parallel algorithms for hierarchical clustering. Parallel computing. 1995;21(8):1313–1325. 10.1016/0167-8191(95)00017-I

[59] Wu CH, Horng SJ, Tsai HR. Efficient parallel algorithms for hierarchical clustering on arrays with reconfigurable optical buses. Journal of Parallel and Distributed Computing. 2000;60(9):1137–1153. 10.1006/jpdc.2000.1644

[60] Kawai H, Garcia-Molina H, Benjelloun O, Menestrina D, Whang E, Gong H. P-swoosh: Parallel algorithm for generic entity resolution. 2006;.

[61] Kim Hs, Lee D. Parallel linkage. In: Proceedings of the sixteenth ACM conference on Conference on information and knowledge management. ACM; 2007. p. 283–292.

[62] Kirsten T, Kolb L, Hartung M, Groß A, Köpcke H, Rahm E. Data partitioning for parallel entity matching arXiv preprint arXiv:10065309. 2010;.

[63] Dal Bianco G, Galante R, Heuser CA. A fast approach for parallel deduplication on multicore processors. In: Proceedings of the 2011 ACM Symposium on Applied Computing. ACM; 2011. p. 1027–1032.

[64] Mi T, Rajasekaran S, Aseltine R. Efficient algorithms for fast integration on large data sets from multiple sources. BMC medical informatics and decision making. 2012;12(1):59 10.1186/1472-6947-12-59 22741525 PMC3439324

[65] Mi T, Aseltine R, Rajasekaran S. Data integration on multiple data sets. In: Bioinformatics and Biomedicine, 2008. BIBM’08. IEEE International Conference on. IEEE; 2008. p. 443–446.

